# Plasma IP-10 and IL-6 are linked to Child-Pugh B cirrhosis in patients with advanced HCV-related cirrhosis: a cross-sectional study

**DOI:** 10.1038/s41598-020-67159-3

**Published:** 2020-06-25

**Authors:** Sergio Salgüero, Luz Maria Medrano, Juan González-García, Juan Berenguer, María L. Montes, Cristina Diéz, Pilar Garcia-Broncano, Elba Llop-Herrera, Leire Pérez-Latorre, José María Bellóno, María Ángeles Jiménez-Sousa, Salvador Resino

**Affiliations:** 1 Unidad de Infección Viral e Inmunidad, Centro Nacional de Microbiología, Instituto de Salud Carlos III, Majadahonda, Madrid Spain; 2 Unidad de Análisis Clínicos, Fundación Hospital Alcorcón, Alcorcón, Madrid Spain; 3 Unidad de VIH; Servicio de Medicina Interna, Hospital Universitario “La Paz”, Madrid, Spain; 4 Unidad de Enfermedades Infecciosas/VIH; Hospital General Universitario “Gregorio Marañón”, Madrid, Spain; 5 Instituto de Investigación Sanitaria del Gregorio Marañón, Madrid, Spain; 6 Ragon Institute of MGH, MIT and Harvard, Cambridge, MA USA; 7 Departamento de Gastroenterología; Hospital Universitario Puerta de Hierro-Majadahonda, Majadahonda, Madrid Spain; 8 Fundación para la Investigación Biomédica, Hospital General Universitario Gregorio Marañón, Instituto de Investigación Sanitaria Gregorio Marañón (IiSGM), Madrid, Spain

**Keywords:** Chemokines, Cytokines, Infectious diseases, Inflammation, Innate immunity, Hepatology, Hepatitis, HIV infections, Viral infection

## Abstract

We aimed to evaluate the association of plasma biomarkers linked to inflammation (bacterial translocation, inflammatory response, and endothelial dysfunction), coagulopathy, and angiogenesis with the severity of liver cirrhosis (assessed by the Child-Pugh-Turcotte score, CTP) and Child-Pugh B cirrhosis (CTP 7–9) in patients with advanced hepatitis C virus (HCV)-related cirrhosis. We carried out a cross-sectional study in 97 patients with advanced HCV-related cirrhosis (32 HCV-monoinfected and 65 HIV/HCV-coinfected). Plasma biomarkers were measured by ProcartaPlex multiplex immunoassays. The outcome variable was the CTP score and the Child-Pugh B cirrhosis (CTP 7–9). HIV/HCV-coinfected patients and HCV-monoinfected patients with advanced HCV-related cirrhosis had near-equivalent values of plasma biomarkers. Higher values of plasma biomarkers linked to an inflammatory response (IP-10, IL-8, IL-6, and OPG), endothelial dysfunction (sVCAM-1 and sICAM-1), and coagulopathy (D-dimer) were related to higher CTP values. The most significant biomarkers to detect the presence of Child-Pugh B cirrhosis (CTP 7–9) were IP-10 (*p*-value= 0.008) and IL-6 (*p*-value=0.002). The AUC-ROC values of IP-10, IL-6, and both biomarkers combined (IP-10+IL-6) were 0.78, 0.88, and 0.96, respectively. In conclusion, HIV infection does not appear to have a significant impact on the analyzed plasma biomarkers in patients with advanced HCV-related cirrhosis. However, plasma biomarkers linked to inflammation (inflammatory response and endothelial dysfunction) were related to the severity of liver cirrhosis (CTP score), mainly IP-10 and IL-6, which discriminated patients with Child-Pugh B concerning Child-Pugh A.

## Introduction

The hepatitis C virus (HCV) has a prevalence of active infection of around 1% worldwide (71 million people)^1^. HCV-infected patients progress slowly during decades (10 to 20 years), developing liver fibrosis and cirrhosis, which can evolve into decompensated cirrhosis and hepatocellular carcinoma^2^. Chronic hepatitis C causes chronic liver inflammation that accelerates the development of cirrhosis and other comorbidities^3,4^. The cirrhosis-associated immune dysfunction (CAID) is a pathophysiological process that appears in cirrhosis and is enhanced in advanced cirrhosis. The CAID is characterized by higher levels of inflammation, immune activation, and deregulation of the immune system, which are related to the progression to hepatic decompensation^5,6^. Patients with hepatic decompensation (Child–Turcotte–Pugh, CTP class B or C) could develop complications related to portal hypertension^2^. Additionally, chronic hepatitis C patients have an increased risk of thrombotic events because hepatocytes produce most blood proteins, and their concentration may be altered during cirrhosis progression^7^.

On the one hand, HCV infection is common among patients infected with human immunodeficiency virus (HIV) infection^8^. HIV/HCV coinfection influences the natural history of chronic hepatitis C, accelerating the progression to cirrhosis and end-stage liver disease in comparison to HCV-monoinfected patients^9,10^. This seems to be due to HIV infection that increases immune activation, HCV replication, HCV-induced hepatic inflammation, hepatocyte apoptosis, and microbial translocation^5,11^, which, in turn, contribute to the pathogenesis of both acquired immunodeficiency syndrome (AIDS) and non-AIDS related diseases^12,13^. Moreover, while suppressive antiretroviral therapy (ART) reduces the HIV impact on the body, it is unable to eradicate the virus. Thus, HIV-infected patients on suppressive ART still show increased bacterial translocation, immune activation, inflammation, and coagulopathy, which are linked to increased morbidity and mortality^12^.

On the other hand, HCV clearance with direct-acting antiviral agents (DAAs) promotes a decrease of liver disease severity and plasma biomarkers linked to bacterial translocation, immune activation, inflammation and coagulopathy in HIV/HCV-coinfected patients^14–18^ and HCV-monoinfected patients^16,17,19,20^. However, a percentage of patients maintain the risk of cirrhosis progression after HCV clearance with DAAs^21,22^. Besides, HIV/HCV-coinfected patients must face drug-drug interactions and hurdles with antiviral treatments, which support that patients coinfected with HIV/HCV might still be regarded as a particular population^23^.

## Objective

We aimed to evaluate the association of plasma biomarkers linked to inflammation (bacterial translocation, inflammatory response, and endothelial dysfunction), coagulopathy, and angiogenesis with the severity of liver cirrhosis (assessed by the CTP score) and Child-Pugh B cirrhosis (CTP 7–9) in patients with advanced HCV-related cirrhosis.

## Patients and methods

### Patients

We carried out a cross-sectional study in 97 patients with advanced HCV-related cirrhosis who were selected from the ESCORIAL cohort (see Acknowledgements), which is a prospective cohort of patients with advanced HCV-related cirrhosis initiating anti-HCV therapy with all-oral DAAs at four tertiary referral hospitals in Madrid, Spain. All patients were enrolled between January and December 2015.

The inclusion criteria of the ESCORIAL cohort were: 1) plasma HCV RNA detectable by polymerase chain reaction; 2) one or more clinical criteria related to advanced cirrhosis (prior history of liver decompensation (ascites, bleeding esophageal varices, hepatic encephalopathy), or liver stiffness measurement (LSM) ≥25 kilopascals (kPa), or hepatic venous pressure gradient (HVPG) ≥10 mmHg, or CTP ≥7); 3) Initiation of all-oral DAA therapy; 4) a biological sample to carry out immunological assays. The exclusion criteria were: i) the previous diagnosis of hepatocellular carcinoma, ii) hepatitis B virus coinfection. The presence of HIV infection was not an exclusion criterion for the study.

In the present study, we only included patients with advanced HCV-related cirrhosis at baseline, when they had not yet started the HCV treatment. The ESCORIAL study included 112 patients, but 15 of them did not have a plasma sample at baseline, leaving only 97 patients available for the study (32 HCV-monoinfected patients and 65 HIV/HCV-coinfected patients.

The ESCORIAL study was conducted according to the Declaration of Helsinki, and the Research Ethics Committee of the Instituto de Salud Carlos III (CEI PI 41_2014) approved this study. Written informed consent was obtained by all the participants in the study.

### Clinical data

Clinical and laboratory data were recorded using a standard database via an online form within each center, which satisfied local requirements of data confidentiality. This process was monitored to verify that all the information in the database was consistent with the patient’s records.

LSM was evaluated by trained operators by transient elastography (FibroScan, Echosens, Paris, France), as we previously described^24^, and results were reported in kPa, with a range of 2.5 to 75 kPa. The CTP score was calculated from five factors (total bilirubin, albumin, international normalized ratio, ascites, and encephalopathy) and range between 5 and 15 points^25^. CTP values serve to classify the patient into one of three severity classes of liver cirrhosis: A – Least severe liver disease (5–6 points), B – Moderately severe liver disease (7–9 points), and C – Most severe derangement (10–15 points). All HIV/HCV-coinfected patients were on ART and had undetectable plasma HIV viral load (<50 copies/mL) at least one year before the study.

### Enzyme-linked immunosorbent assays

The Spanish HIV HGM BioBank collected plasma samples, which were stored until use at –80 °C. We evaluated plasma biomarkers by ProcartaPlex multiplex immunoassay (Bender MedSystems GmbH, Vienna, Austria) according to the manufacturer’s specifications using a Luminex 200 analyzer (Luminex Corporation, Austin, TX, United States). The plasma biomarkers measured by multiplex ELISA were: i) inflammatory response: IFN-γ-inducible protein 10 (IP-10), monocyte chemoattractant protein-1 (MCP1), IL-8, IL-1β, IL-18, IL-6, tumor necrosis factor-alpha (TNF-α), interleukin-1 receptor antagonist (IL-1RA), soluble receptor activator of nuclear factor- kappaB ligand (sRANKL) and osteoprotegerin (OPG); ii) endothelial dysfunction: soluble vascular cell adhesion molecule 1 (sVCAM-1), soluble intercellular cell adhesion molecule 1 (sICAM-1); and soluble tumor necrosis factor receptor 1 (sTNF-R1); iii) coagulopathy: plasminogen activator inhibitor-1 (PAI-1) and d-dimer; iv) angiogenesis/fibrosis: vascular endothelial growth factor A (VEGF-A) and soluble receptors for vascular endothelial growth factor (sVEGF-R1). In these assays, a high proportion of the analyzed samples were below the lower limit of detection (LOD), and the analysis software censored calculated biomarker levels. The measured fluorescence intensity (FI) values are an alternative to alleviate the concern of determining levels of LOD. Because of this, we did the data analysis using the raw FI values, without subtracting blank, as a relative quantification of the analyte abundances^26^. With this approach, there were no missing values, it was not necessary to specify a LOD, and we can analyze the low FI signals, which added more statistical power to the data analysis^26,27^. All measured FI (arbitrary units, a.u.) values were normalized using log10 transformation (log10 transformed).

The plasma biomarkers measured by simple ELISA were lipopolysaccharide-binding protein (LBP; (R&D Systems, Minneapolis, USA), sCD14, and fatty acid-binding protein 2 (FABP-2) (Raybiotech, Georgia, USA)). The lipopolysaccharide was evaluated by a *Limulus* amebocyte lysate chromogenic endpoint ELISA (LPS; Hycult Biotech, Uden, The Netherlands).

### Statistical analysis

The statistical analysis was performed with Stata 15.0 (StataCorp, Texas, USA) and Statistical Package for the Social Sciences (SPSS) 22.0 (SPSS INC, Chicago, IL, USA). All *p*-values were two-tailed, and statistical significance was defined as *p* < 0.05.

For the descriptive analysis, categorical variables were analyzed by the chi-squared test or Fisher’s exact test, as required, and the Mann-Whitney test was used to analyze continuous data.

In this study, the outcome variables were the severity of liver cirrhosis, evaluated with the CTP score, and the presence of severe cirrhosis with Child-Pugh B (CTP 7–9). For the statistical association analysis, Generalized Linear Models (GLM) with a gamma distribution (log-link) were used to analyze the relationship among plasma biomarkers and the CTP score. Besides, GLM with binomial distribution was used to analyze the differences in plasma biomarkers between study groups. These tests give us: i) the arithmetic mean ratio (AMR) and the odds ratio (OR), and ii) significance levels (*p*-values), which were corrected for multiple testing using the false discovery rate (*FDR*) with Benjamini and Hochberg (*q*-values) procedure to reduce the risk of spurious results. GLM models were also adjusted by clinical and epidemiological co-variables: age, gender, smoker, alcohol intake, intravenous drug user (IVDU), previous IFNα therapy, statins treatment, HCV genotype, and log_10_ HCV RNA. Each plasma biomarker was included by forced entry (Enter algorithm), and the most significant co-variables were selected by a stepwise algorithm (at each step, co-variables are considered for entry with a p-value <0.20), allowing to avoid the over-fitting of the regression.

The accuracy of the biomarkers to separate the study groups was evaluated by the area under the ROC curve (AUC-ROC). Youden’s index was used to select the best cut-off.

### Ethics approval and consent to participate

The study was conducted in accordance with the Declaration of Helsinki and patients gave their written consent. The Institutional Review Board and the Research Ethic Committee of the Instituto de Salud Carlos III (ISCIII) approved the study.

## Results

### Patients

The characteristics of the 97 patients with advanced HCV-related cirrhosis (32 HCV-monoinfected patients and 65 HIV/HCV-coinfected patients) are shown in Table 1. HIV/HCV-coinfected patients had the lowest values of age (*p*-value <0.001) and CTP score (*p*-value= 0.012), lower percentages of previously treated with IFNα therapy (*p*-value= 0.037), and Child-Pugh B cirrhosis (CTP 7–9) (*p*-value= 0.009); while they had the highest percentages of males (*p*-value= 0.037) and IVDUs (*p*-value ≤0.001). The percentage of prior history of liver decompensation was similar in both groups (p = 0.375). Among patients with Child-Pugh A cirrhosis (CTP < 7), 11 had LSM < 25 kPa, and five had HVPG < 10 mmHg. All patients with Child-Pugh B cirrhosis (CTP 7–9) had LSM ≥25 kPa and HVPG ≥10 mmHg. No patients were in Child-Pugh C cirrhosis.

**Table 1:** Summary of epidemiological and clinical characteristics in HCV-monoinfected patients and HIV/HCV-coinfected patients who had advanced HCV-related cirrhosis.

	All	HCV	HIV/HCV	*p*-values
No.	97	32	65	
Gender (male) (n = 97)	68 (70.1%)	18 (56.3%)	50 (76.9%)	**0.037**
Age (years) (n = 97)	53 (48.8; 56.5)	59.5 (52.6; 70)	51.8 (48.7; 53.8)	**<0.001**
**Smoker (n = 96)**				
Never	19 (19.8%)	11 (34.4%)	8 (12.5%)	0.589
Previously (≥ 6 months)	25 (26%)	10 (31.3%)	15 (23.4%)	0.988
Nowadays	52 (54.2%)	11 (34.4%)	41 (64.1%)	0.153
**Alcohol intake (n = 97)**				
Never	49 (50.5%)	22 (68.8%)	27 (41.5%)	0.105
Previously (≥ 6 months)	40 (41.2%)	9 (28.1%)	31 (47.7%)	0.507
Nowadays	8 (8.2%)	1 (3.1%)	7 (10.8%)	0.120
IVDU (n = 97)				
Never	40 (41.2%)	27 (84.4%)	13 (20%)	**<0.001**
Previously (≥ 6 months)	57 (58.8%)	5 (15.6%)	52 (80%)	**0.009**
Current	0 (0%)	0 (0%)	0 (0%)	—
**Treatments**				
Previous IFNα therapy (n = 97)	49 (50.5%)	21 (65.6%)	28 (43.1%)	**0.037**
Statins (n = 97)	11 (11.3%)	1 (3.1%)	10 (15.4%)	0.073
**Antiretroviral therapy (n = 65)**				
NRTI + NNRTI-based	—	—	7 (10.8%)	
NRTI + II-based	—	—	34 (52.3%)	
NRTI + PI-based	—	—	9 (13.8%)	
PI + II + others-based	—	—	4 (6.2%)	
Others	—	—	11 (16.9%)	
**HIV markers**				
Prior AIDS (n = 65)	—	—	23 (35.4%)	
Nadir CD4 + T-cells (cells/mm3) (n = 60)	—	—	129 (70; 243.5)	
Nadir CD4 + < 200 cells/mm3 (n = 60)	—	—	40 (66.7%)	
CD4 + T-cells (cells/mm3) (n = 65)	—	—	444 (234; 719)	
CD4 + < 500 cells/mm3 (n = 65)	—	—	38 (58.5%)	
Undetectable HIV-RNA (n = 65)	—	—	65 (100%)	
**HCV markers**				
HCV genotype (n = 95)				
1	65 (68.4%)	25 (78.1%)	40 (63.5%)	0.335
3	14 (14.7%)	4 (12.5%)	10 (15.9%)	0.503
4	16 (16.8%)	3 (9.4%)	13 (20.6%)	0.708
Log_10_ HCV-RNA (IU/mL) (n = 96)	6.1 (5.5; 6.5)	6.1 (5.4; 6.4)	6.2 (5.6; 6.6)	0.287
HCV-RNA ≥ 850.000 IU/mL	60 (62.5%)	19 (59.4%)	41 (64.1%)	0.655
**Liver disease (n = 94)**				
LSM (kPa)	30.6 (24.5; 41.6)	29.9 (26.3; 48)	31 (22.3; 39.3)	0.208
<25 kPa	24 (25.5%)	6 (19.4%)	18 (28.6%)	0.926
25–40 kPa	45 (47.9%)	14 (45.2%)	31 (49.2%)	0.941
≥40 kPa	25 (26.6%)	11 (35.5%)	14 (22.2%)	0.774
Child-Pugh Score (n = 91)	5 (5; 6)	5 (5; 7)	5 (5; 5)	**0.012**
Child-Pugh B (7–9)	13 (14.3%)	8 (28.6%)	5 (7.9%)	**0.009**
Prior history of liver decompensation	30 (31%)	8 (22.8%)	22 (33.8%)	0.375

Statistics: Values expressed as absolute number (percentage) and median (interquartile range). P-values were calculated by Chi-square, Fisher’s exact test, and Mann-Whitney tests, as required. The statistically significant differences are shown in bold.

**Abbreviations**: HCV, hepatitis C virus; HIV, human immunodeficiency virus; IVDU, intravenous drug user; IFNα, interferon-alpha; NRTI, nucleoside analogue HIV reverse; NNRTI, non-nucleoside analogue HIV reverse transcriptase inhibitor; PI, protease inhibitor; II, integrase inhibitor; AIDS, acquired immune deficiency syndrome; HIV-RNA, HIV plasma viral load; HCV-RNA, HCV plasma viral load; LSM, liver stiffness measure; kPa, kilopascal.

### HIV/HCV-coinfected patients vs. HCV-monoinfected patients

HIV/HCV-coinfected patients had lower values of sCD14 (p-value= 0.046), IL-1RA (*p*-value= 0.049) and sRANKL (p-value= 0.019) than HCV-monoinfected patients in adjusted regression analyses. However, all these significant differences disappeared after adjusting by multiple comparisons (see Supplementary Table 1). Therefore, HIV/HCV-coinfected patients on suppressive ART had quite similar values of plasma biomarkers than HCV-monoinfected patients.

### Relationship between plasma biomarkers and CTP score

**Figure 1:**
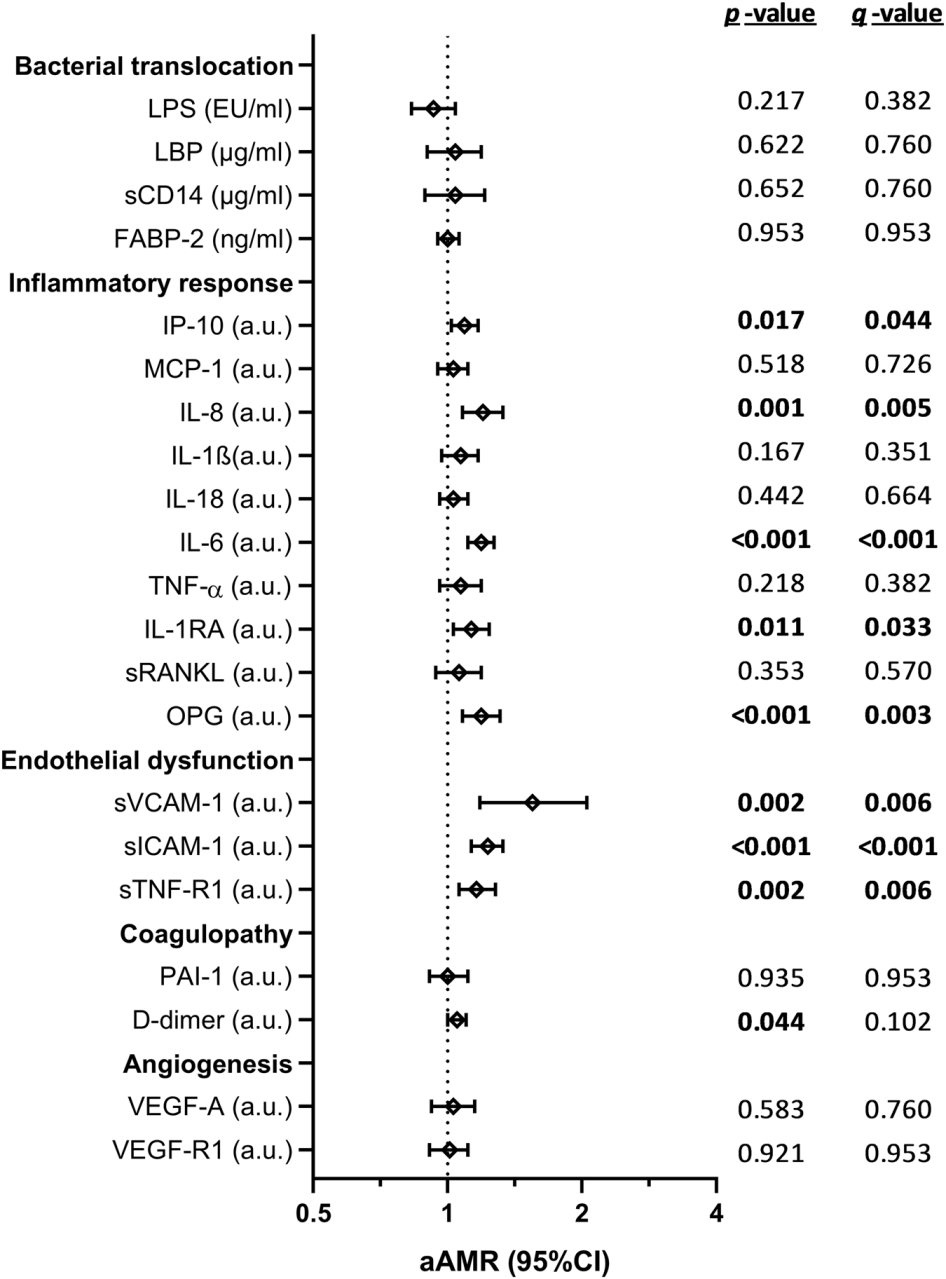
Association between values of plasma biomarkers (fluorescence intensity, arbitrary units) and Child-Pugh-Turcotte (CTP) score in patients with advanced HCV-related cirrhosis. **Statistics**: Values were expressed as arithmetic mean ratio (aAMR) and 95% confidence interval (95%CI). *P-values* were calculated by GLM models unadjusted and adjusted by the main clinical and epidemiological characteristics (see statistical analysis section). *P-values*, raw *p*-values; *q*-values, *p*-values corrected for multiple testing using the false discovery rate (*FDR*) with Benjamini and Hochberg procedure. The statistically significant differences are shown in bold. **Abbreviations**: HCV, hepatitis C virus; -1, human immunodeficiency virus type 1; a.u., arbitrary units of fluorescence; sCD14, soluble CD14; LPS, lipopolysaccharide; FABP2, fatty acid-binding protein 2; LBP, lipopolysaccharide binding protein; IL, interleukin; IL-1RA, interleukin-1 receptor antagonist; TNF-α, tumor necrosis factor alpha; IP-10, IFN-γ-inducible protein 10; MCP1, monocyte chemoattractant protein-1; OPG, osteoprotegerin; sRANKL, soluble receptor activator of nuclear factor- kappaB ligand; sVCAM-1, soluble vascular cell adhesion molecule 1; sICAM-1, soluble intercellular cell adhesion molecule 1; sTNF-R1, soluble tumor necrosis factor receptor 1; PAI-1, plasminogen activator inhibitor-1; VEGF-A; vascular endothelial growth factor A; sVEGF-R1, soluble receptors for vascular endothelial growth factor.

Figure 1 shows the adjusted association values between plasma biomarkers and CTP score (full description in Supplementary Table 2). For all patients, higher values of plasma biomarkers linked to inflammatory response [IP-10 (*q*-value= 0.044), IL-8 (*q*-value= 0.005), IL-6 (*q*-value <0.001), and OPG (*q*-value= 0.003)],endothelial dysfunction [sVCAM-1 (*q*-value= 0.006), sICAM-1 (*q*-value <0.001), and sTNF-R1 (*q*-value= 0.006)] were related to higher CTP values. When patients were stratified by HIV infection, HCV-monoinfected patients had association with CTP score for IL-8 (*q*-value= 0.001), IL-6 (*q*-value= 0.002), OPG (*q*-value= 0.001), sVCAM-1 (*q*-value <0.001), sICAM-1 (*q*-value <0.001), and sTNF-R1 (*q*-value= 0.002); while HIV/HCV-coinfected patients only showed significant association with CTP score for IL-6 (*q*-value= 0.007) and sICAM-1 (*q*-value= 0.007).

**Figure 2:**
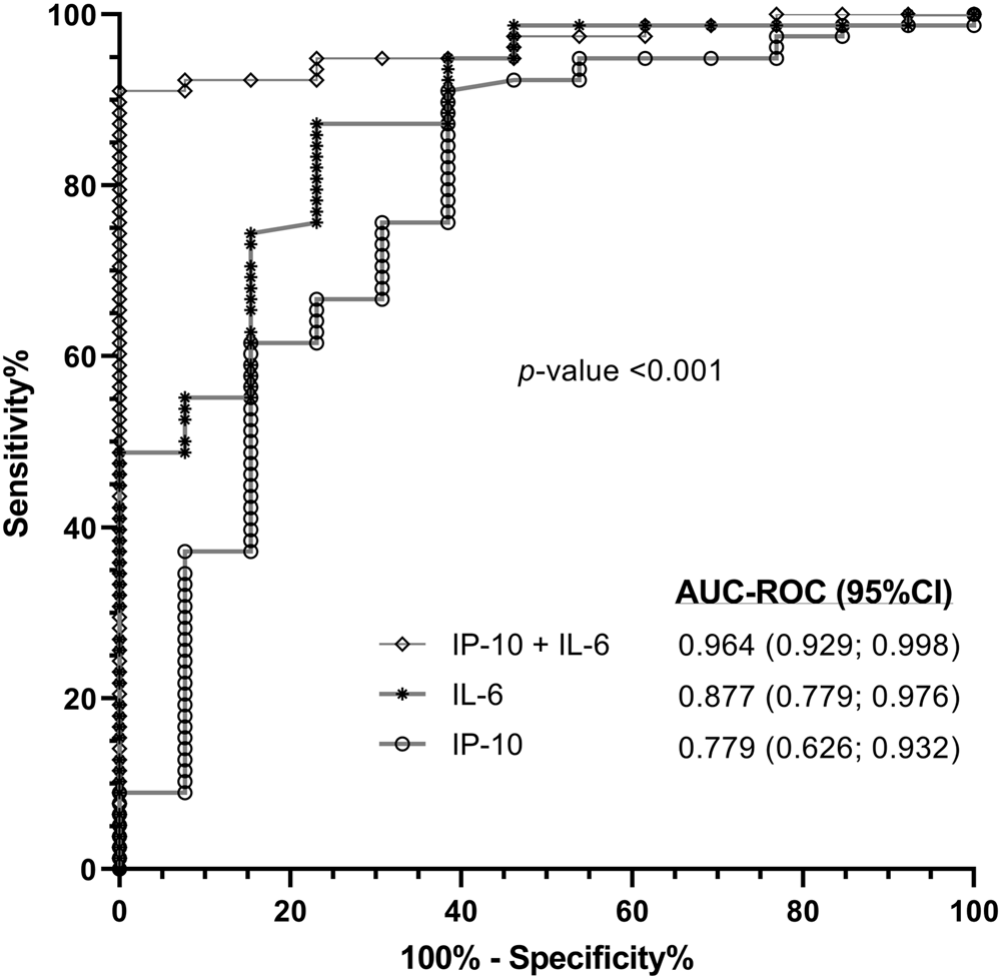
Receiver operating characteristic (ROC) curves of plasma biomarkers for predicting Child-Pugh B cirrhosis (CTP 7–9) in patients with advanced HCV-related cirrhosis. **Statistics**: Values were expressed as area under the receiver operating characteristic (AUC-ROCs) and 95% confidence interval (95%CI). **Abbreviations**: IP-10, IFN-γ-inducible protein 10; IL-6, interleukin 6.

Patients with Child-Pugh B cirrhosis (CTP 7–9) had higher plasma values of IP-10, IL-6, OPG, sVCAM-1, sICAM-1, and D-dimer (*p*-value <0.05 and *q*-value <0.1) than patients with Child-Pugh A cirrhosis (CTP < 7) (Table 2). The AUC-ROC values of these biomarkers to separate the study groups (CTP < 7 vs CTP 7–9) were higher than 0.70 and significant for IP-10 (*q*-value= 0.012), IL-6 (*q*-value <0.001), OPG (*q*-value= 0.012), sVCAM-1 (*q*-value= 0.036), sICAM-1 (*q*-value= 0.023), and D-dimer (*q*-value= 0.036) (Table 2). We also selected, by a multivariate logistic regression with stepwise algorithm, IP-10 (*p*-value= 0.008) and IL-6 (*p*-value= 0.002) as the most significant biomarkers. The AUC-ROC of IP-10, IL-6, and both biomarkers combined (IP-10+IL-6) were 0.78, 0.88, and 0.96; respectively (Fig. 2). The value of 0.15 of IP-10 and IL-6 combined in a logistic regression model was the best cut-off (Youden’s index = 0.846), which showed a sensitivity and specificity of 92.3%, positive predictive value of 98.6% and negative predictive value of 66.7%.

**Table 2:** Summary of plasma biomarkers (fluorescence intensity, arbitrary units) in patients with advanced HCV-related cirrhosis according to CTP score.

	All patients		GLM (binomial)		ROC curve		*q*-value
	CTP < 7	CTP ≥ 7	*p*-value	*q*-value	AUC-ROC (95%CI)	*p*-value	
**Bacterial translocation**							
LPS (EU/ml)	1.04 (0.81; 1.51)	0.92 (0.68; 1.23)	0.124	0.258	0.39 (0.23; 0.56)	0.214	0.281
LBP (µg/ml)	0.97 (0.67; 1.28)	0.83 (0.69; 1.34)	0.667	0.778	0.5 (0.32; 0.68)	0.991	0.991
sCD14 (µg/ml)	2.23 (1.72; 3.08)	3.26 (2.19; 3.8)	0.135	0.258	0.65 (0.49; 0.82)	0.075	0.169
FABP-2 (ng/ml)	0.47 (0.23; 0.91)	0.65 (0.32; 1.25)	0.787	0.870	0.56 (0.39; 0.74)	0.478	0.558
**Inflammatory response**							
IP-10 (a.u.)	1077.7 (724; 1573)	2218.5 (1340; 2551.5)	**0.005**	**0.035**	0.78 (0.63; 0.93)	**0.001**	**0.012**
MCP-1 (a.u.)	427 (205.5; 651.5)	553.5 (294; 714.5)	0.316	0.474	0.58 (0.41; 0.74)	0.373	0.461
IL-8 (a.u.)	100 (64; 147.5)	154 (81; 220)	0.054	0.142	0.65 (0.49; 0.82)	0.081	0.169
IL-1β (a.u.)	15 (14; 22)	17.5 (15; 28)	0.277	0.447	0.66 (0.5; 0.81)	0.067	0.169
IL-18 (a.u.)	857.5 (496; 1536)	1024 (541; 1216)	0.973	0.973	0.48 (0.34; 0.62)	0.829	0.871
IL-6 (a.u.)	68.25 (36; 129)	265 (167.5; 360)	**<0.001**	**0.006**	0.88 (0.78; 0.98)	**<0.001**	**<0.001**
TNF-α (a.u.)	10 (7.5; 12)	11 (9; 14)	0.493	0.610	0.63 (0.48; 0.78)	0.140	0.227
IL-1RA (a.u.)	37.5 (31; 51)	62 (42; 77)	0.437	0.574	0.67 (0.53; 0.82)	**0.049**	0.147
sRANKL (a.u.)	25 (20; 35)	36 (24.5; 58)	**0.048**	0.142	0.64 (0.44; 0.84)	0.102	0.179
OPG (a.u.)	188 (135.5; 277)	347.5 (288; 486)	**0.016**	0.066	0.76 (0.63; 0.9)	0.002	0.012
**Endothelial dysfunction**							
sVCAM-1 (a.u.)	10482 (8946; 11558.5)	11956.5 (10603; 12554.5)	**0.019**	0.066	0.72 (0.59; 0.86)	**0.010**	**0.036**
sICAM-1 (a.u.)	87.75 (59; 130)	198 (108.5; 288.5)	**0.002**	**0.023**	0.77 (0.63; 0.91)	**0.002**	**0.012**
TNF-R1 (a.u.)	28.75 (19; 41)	38 (31; 57.5)	0.186	0.326	0.62 (0.47; 0.78)	0.156	0.234
**Coagulopathy**							
PAI-1 (a.u.)	1026 (812; 1287)	955 (815; 1187)	0.883	0.927	0.48 (0.3; 0.65)	0.794	0.871
D-dimer (a.u.)	1632.7 (602; 3825.5)	5443 (4542.5; 7582)	**0.017**	0.066	0.73 (0.58; 0.87)	**0.009**	**0.036**
**Angiogenesis/Fibrosis**							
VEGF-A (a.u.)	75.5 (55.5; 106)	88 (72; 95)	0.437	0.574	0.61 (0.47; 0.76)	0.192	0.269
VEGF-R1 (a.u.)	44 (35; 68)	52 (44.5; 129)	0.104	0.243	0.65 (0.47; 0.82)	0.093	0.178

Statistics: Values expressed as median (P25th; P75th), area under the ROC curve (AUC-ROC), and 95% of confidence interval (95%CI). P-values were calculated by GLMs adjusted by the main clinical and epidemiological characteristics (see statistical analysis section). P-values, raw p-values; q-values, p-values corrected for multiple testing using the false discovery rate (FDR) with Benjamini and Hochberg procedure. The statistically significant differences are shown in bold.

**Abbreviations**: HCV, hepatitis C virus; HIV, human immunodeficiency virus; a.u., arbitrary units of fluorescence; sCD14, soluble CD14; LPS, lipopolysaccharide; FABP2, fatty acid-binding protein 2; LBP, lipopolysaccharide binding protein; IL, interleukin; IL-1RA, interleukin-1 receptor antagonist; TNF-α, tumor necrosis factor alpha; IP-10, IFN-γ-inducible protein 10; MCP1, monocyte chemoattractant protein-1; OPG, osteoprotegerin; sRANKL, soluble receptor activator of nuclear factor- kappaB ligand; sVCAM-1, soluble vascular cell adhesion molecule 1; sICAM-1, soluble intercellular cell adhesion molecule 1; sTNF-R1, soluble tumor necrosis factor receptor 1; PAI-1, plasminogen activator inhibitor-1; VEGF-A; vascular endothelial growth factor A; sVEGF-R1, soluble receptors for vascular endothelial growth factor.

## Discussion

In this study, we have evaluated the profile of plasma biomarkers (inflammation, endothelial dysfunction, coagulopathy, and angiogenesis) in patients with advanced HCV-related cirrhosis. We found that HIV/HCV-coinfected patients on suppressive ART and HCV-monoinfected patients had near-equivalent values of plasma biomarkers. We also found that higher values of plasma biomarkers (IP-10, IL-8, IL-6, OPG, sVCAM-1, sICAM-1, and D-dimer) were related to higher values of liver disease severity (CTP), but only IP-10 and IL-6 had high accuracy in separating patients with Child-Pugh B cirrhosis (CTP 7–9).

HIV/HCV-coinfected patients usually have a faster progression of chronic hepatitis C^28^ and higher levels of plasma biomarkers of bacterial translocation, immune activation, inflammation, and coagulation, despite suppressive ART^29^. In our study, plasma values of biomarkers were very similar in HIV/HCV-coinfected and HCV-monoinfected patients with advanced HCV-related cirrhosis. This may be because our patients had advanced cirrhosis, where elevated immune activation, inflammation, and dysregulation of the innate immune system is usually present^5,6^. Thus, we could suggest that the weight of cirrhosis was so important that the impact of HIV infection, if any, was eclipsed. Another argument could be that HIV/HCV-coinfected patients were on suppressive ART, with long-term optimal control of HIV replication and significant immune recovery, as shown by the difference between the values of CD4^+^ T-cells nadir and current count.

Both HIV and HCV infections are characterized by an increased inflammatory response, which raises as the severity of the liver disease progresses^4,24,30^. Inflammation is also linked to endothelial dysfunction, which is related to the higher severity of liver disease^31^. Besides, inflammation (inflammatory response and endothelial dysfunction) is associated with increased risk of AIDS progression in HIV-infected patients, the development of comorbidities, chronic hepatitis C progression, and death^30,32–34^. In our study, we found a significant association between plasma biomarkers linked to inflammation [inflammatory response (IP-10, IL-8, IL-6, and OPG) and endothelial dysfunction (sVCAM-1 and sICAM-1)] and CTP score in all patients with advanced HCV-related cirrhosis so that the highest levels of these biomarkers were found in patients with greater severity of cirrhosis, suggesting a more pronounced inflammatory CAID phenotype, which is in line with previously published data in patients with severe cirrhosis^5,6^. Moreover, a large number of blood proteins are produced in the liver, and their blood levels may be altered in advanced stages of cirrhosis, leading to increased thrombotic risk^7^. Coagulopathy is related to increased risk of disease progression and death in people infected with HIV^35^ and HCV^36^. In this study, we found a significant association between D-dimer and the CTP score in all patients, but this disappeared when the population was stratified by HIV-infection, possibly because the association was affected by the decreased sample size when the sample was stratified by HIV infection. In any case, there are indications that higher levels of D-dimer are found in patients with a greater stage of cirrhosis. Besides systemic inflammation, the main reason for elevated D-dimer levels in decompensated cirrhosis (more precisely, patients with ascites) is systemic hyperfibrinolysis due to the intraperitoneal activation of the coagulation cascade by tissue factor-bearing extracellular vesicles^37^.

In our study, HIV infection seems to have had a significant impact on the association between plasma biomarkers and the CTP score. However, the associations had the same sense in the two study groups, although they were weaker in HIV/HCV-coinfected patients, possibly because CTP values had a narrower range in HIV/HCV-coinfected patients than in HCV-monoinfected patients. Thereby, HCV-monoinfected patients showed a significant association of plasma biomarkers of the inflammatory response (IL-8, IL-6, and OPG) and endothelial dysfunction (sVCAM-1, sICAM-1, and sTNF-R1) with CTP score; while HIV/HCV-coinfected patients showed only a significant association of IL-6 and sICAM-1 with CTP score. It is therefore complicated to state that, in our study, HIV infection had a relevant impact on the relationship between biomarkers and the CTP score; nevertheless, it seems clear that the association between inflammation and CTP score remains, independently of HIV infection.

High levels of inflammation is a feature of patients with advanced CAID, particularly in patients with hepatic decompensation^5,6^. In our study, we found patients who had Child-Pugh B cirrhosis (CTP 7–9) showed higher plasma values of biomarkers linked to the inflammatory response (IP-10, IL-6, and OPG) endothelial dysfunction (sVCAM-1 and sICAM-1) and coagulopathy (D-dimer). These biomarkers were practically the same as those discussed in the previous paragraphs. However, only IP-10 and IL-6 were independently associated with Child-Pugh B cirrhosis (CTP 7–9) with high accuracy. Increased plasma IL-6 and IP-10 levels are related to liver disease severity in HCV-infected patients^11,38^ and HIV/HCV-coinfected patients^24^, but in the current study, we found plasma IL-6 and IP-10 levels discriminated with great accuracy the presence of severe cirrhosis with Child-Pugh B (CTP 7–9). The severe cirrhosis seems to be the result of an inflammatory syndrome, which increases the risk of acute-on-chronic liver failure (ACLF)^39^. Besides, systemic inflammation could also be implicated in the pathogenesis of extrahepatic organ dysfunctions^39^. However, severe cirrhosis is easy to detect in the clinical setting by physical examination and abdominal ultrasound. This means the use of inflammatory serological markers to identify patients with Child-Pugh B (CTP 7–9) may be of little use in the clinic. Despite this, the AUROC analysis provides helpful information for these two significant inflammatory biomarkers (IL-6 and IP-10), because these two biomarkers also show distinctive elevated levels in Child-Pugh B (CTP 7–9) compared to Child-Pugh A (CTP 5–6), which can aid proper classification.

## Limitations of the study

Firstly, this is a cross-sectional study with a low sample size, which may entail a lack of uniformity and could limit the possibility of finding statistical significance in some subgroups. However, despite this, we systematically find inflammation biomarkers associated with the CTP score values, which gives consistency to our results. Furthermore, the potential clinical translation may be limited by the cross-sectional design of our study. A different research design would be necessary to determine if the possible pathogenic factors evaluated in our study are involved in the progression of the liver disease since it cannot be revealed by a cross-sectional approach that does not describe the course of the disease over time. Secondly, all selected patients met a set of criteria for our study, and this may have introduced a selection bias. Longitudinal studies with a higher number of subjects, and with less restrictive criteria, would be necessary to generalize our conclusions to patients with advanced HCV-related cirrhosis and whether the IP-10 and/or IL-6 could predict the patients progressing from CTP A to CTP B.

## Conclusions

Plasma biomarker values were quite similar in HIV/HCV-coinfected patients on suppressive ART and HCV-monoinfected patients. Besides, values of plasma biomarkers linked to inflammation (inflammatory response and endothelial dysfunction) were related to the severity of liver cirrhosis (CTP score), mainly IP-10 and IL-6, which discriminated patients with Child-Pugh B concerning Child-Pugh A.

## Supplementary information

Supplementary Information.

Supplementary Information2.

## Data Availability

The datasets used and/or analyzed during the current study may be available from the corresponding author upon reasonable request.
