# Supplementary material for: Plasma IP-10 and IL-6 are linked to Child-Pugh B cirrhosis in patients with advanced HCV-related cirrhosis: a cross-sectional study
Source: Sci Rep. 2020 Jun 25;10:10384. doi: 10.1038/s41598-020-67159-3 (PMC7316790; doi:10.1038/s41598-020-67159-3)
Supplement: Supplementary file 2 — Supplementary Information2. [file 41598_2020_67159_MOESM2_ESM.docx]

**Type of manuscript:** Original article

**Title**: Plasma IP-10 and IL-6 are linked to Child-Pugh B cirrhosis in patients with advanced HCV-related cirrhosis: a cross-sectional study

**Running head**: Inflammation and severity of cirrhosis

**Authors:** Sergio SALGÜERO ^1, 2, ¥^, Ms.C.; Luz Maria MEDRANO ^1^, Ph.D. ^¥^; Juan GONZÁLEZ-GARCÍA ^3^, M.D., Ph.D.; Juan BERENGUER ^4, 5^, M.D., Ph.D.; María L. MONTES ^3^, M.D., Ph.D.; Cristina DÍEZ ^4, 5^, M.D., Ph.D.; Pilar GARCIA-BRONCANO ^1,6^, Ph.D.; Elba LLOP-HERRERA ^7^, M.D., Ph.D.; Leire PÉREZ-LATORRE ^4,5^, M.D., Ph.D.; José María BELLÓN ^8^, Bs.C.; Mª Ángeles JIMÉNEZ-SOUSA ^1, *^, Ph.D.; Salvador RESINO ^1, *^, Ph.D.

(¥) Both authors contributed equally to this study; (*) Corresponding authors.

**Authors’ affiliations:**

(1) Unidad de Infección Viral e Inmunidad, Centro Nacional de Microbiología, Instituto de Salud Carlos III, Majadahonda, Madrid, Spain.

(2) Unidad de Análisis Clínicos, Fundación Hospital Alcorcón, Spain.

(3), Unidad de VIH; Servicio de Medicina Interna, Hospital Universitario “La Paz”, Madrid, Spain.

(4) Unidad de Enfermedades Infecciosas/VIH; Hospital General Universitario “Gregorio Marañón”, Madrid, Spain.

(5) Instituto de Investigación Sanitaria del Gregorio Marañón, Madrid, Spain.

(6) Ragon Institute of MGH, MIT and Harvard, Cambridge, MA, USA

(7) Departamento de Gastroenterología; Hospital Universitario Puerta de Hierro-Majadahonda; Majadahonda, Madrid; Spain

(8) Fundación para la Investigación Biomédica, Hospital General Universitario Gregorio Marañón, Instituto de Investigación Sanitaria Gregorio Marañón (IiSGM), Madrid, Spain

**Supplementary Table 2**. Association between values of plasma biomarkers (fluorescence intensity, arbitrary units) and Child-Pugh-Turcotte (CTP) score in patients with advanced HCV-related cirrhosis.

|  | **All patients** | | | | | |
| --- | --- | --- | --- | --- | --- | --- |
|  | **Unadjusted GLM** | | | **Adjusted GLM** | | |
|  | **AMR (95%CI)** | ***p*-value** | ***q*-value** | **AMR (95%CI)** | ***p*-value** | ***q*-value** |
| **Bacterial translocation** |  |  |  |  |  |  |
| LPS (EU/ml) | 0.93 (0.82; 1.06) | 0.261 | 0.375 | 0.93 (0.83; 1.04) | 0.217 | 0.382 |
| LBP (µg/ml) | 1.02 (0.87; 1.19) | 0.840 | 0.840 | 1.04 (0.9; 1.19) | 0.622 | 0.760 |
| sCD14 (µg/ml) | 1.12 (0.96; 1.3) | 0.159 | 0.261 | 1.04 (0.89; 1.21) | 0.652 | 0.760 |
| FABP-2 (ng/ml) | 1.03 (0.97; 1.08) | 0.339 | 0.459 | 1 (0.95; 1.06) | 0.953 | 0.953 |
| **Inflammatory response** |  |  |  |  |  |  |
| IP-10 (a.u.) | 1.12 (1.04; 1.21) | **0.004** | **0.020** | 1.09 (1.02; 1.17) | **0.017** | **0.044** |
| MCP-1 (a.u.) | 1.04 (0.95; 1.13) | 0.435 | 0.553 | 1.03 (0.95; 1.11) | 0.518 | 0.726 |
| IL-8 (a.u.) | 1.17 (1.05; 1.31) | **0.006** | **0.022** | 1.2 (1.08; 1.33) | **0.001** | **0.005** |
| IL-1β (a.u.) | 1.12 (1.02; 1.23) | **0.024** | 0.061 | 1.07 (0.97; 1.17) | 0.167 | 0.351 |
| IL-18 (a.u.) | 1.02 (0.94; 1.1) | 0.641 | 0.702 | 1.03 (0.96; 1.11) | 0.442 | 0.664 |
| IL-6 (a.u.) | 1.21 (1.13; 1.3) | **<0.001** | **<0.001** | 1.19 (1.11; 1.27) | **<0.001** | **<0.001** |
| TNF-α (a.u.) | 1.11 (0.99; 1.24) | 0.079 | 0.140 | 1.07 (0.96; 1.19) | 0.218 | 0.382 |
| IL-1RA (a.u.) | 1.12 (1.01; 1.25) | **0.037** | 0.078 | 1.13 (1.03; 1.24) | **0.011** | **0.033** |
| sRANKL (a.u.) | 1.14 (1.01; 1.29) | **0.031** | 0.071 | 1.06 (0.94; 1.19) | 0.353 | 0.570 |
| OPG (a.u.) | 1.19 (1.07; 1.32) | **0.002** | **0.013** | 1.19 (1.08; 1.31) | **<0.001** | **0.003** |
| **Endothelial dysfunction** |  |  |  |  |  |  |
| sVCAM-1 (a.u.) | 1.49 (1.09; 2.02) | **0.012** | **0.040** | 1.55 (1.18; 2.05) | **0.002** | **0.006** |
| sICAM-1 (a.u.) | 1.22 (1.11; 1.34) | **<0.001** | **<0.001** | 1.23 (1.13; 1.33) | **<0.001** | **<0.001** |
| TNFR-I (a.u.) | 1.11 (1.01; 1.24) | **0.049** | 0.095 | 1.16 (1.06; 1.28) | **0.002** | **0.006** |
| **Coagulopathy** |  |  |  |  |  |  |
| PAI-1 (a.u.) | 1.01 (0.9; 1.14) | 0.833 | 0.840 | 1 (0.91; 1.11) | 0.935 | 0.953 |
| D-dimer (a.u.) | 1.08 (1.02; 1.13) | **0.004** | **0.020** | 1.05 (1; 1.1) | **0.044** | 0.102 |
| **Angiogenesis/Fibrosis** |  |  |  |  |  |  |
| VEGF-A (a.u.) | 1.05 (0.93; 1.19) | 0.457 | 0.553 | 1.03 (0.92; 1.15) | 0.583 | 0.760 |
| VEGF-R1 (a.u.) | 1.07 (0.97; 1.19) | 0.174 | 0.266 | 1.01 (0.91; 1.11) | 0.921 | 0.953 |
|  |  |  |  |  |  |  |
|  | **HCV-infected patients** | | | | | |
|  | **Unadjusted GLM** | | | **Adjusted GLM** | | |
|  | **AMR (95%CI)** | ***p*-value** | ***q*-value** | **AMR (95%CI)** | ***p*-value** | ***q*-value** |
| **Bacterial translocation** |  |  |  |  |  |  |
| LPS (EU/ml) | 1.07 (0.72; 1.58) | 0.743 | 0.790 | 0.9 (0.61; 1.33) | 0.588 | 0.650 |
| LBP (µg/ml) | 0.74 (0.46; 1.21) | 0.231 | 0.348 | 0.61 (0.4; 0.94) | **0.025** | 0.074 |
| sCD14 (µg/ml) | 1.45 (0.95; 2.19) | 0.083 | 0.235 | 1.33 (0.91; 1.95) | 0.138 | 0.291 |
| FABP-2 (ng/ml) | 1.03 (0.86; 1.22) | 0.780 | 0.790 | 1.09 (0.93; 1.27) | 0.286 | 0.427 |
| **Inflammatory response** |  |  |  |  |  |  |
| IP-10 (a.u.) | 1.3 (1.08; 1.56) | **0.005** | **0.015** | 1.21 (1.02; 1.44) | **0.031** | 0.082 |
| MCP-1 (a.u.) | 1.12 (0.94; 1.34) | 0.207 | 0.341 | 1.08 (0.92; 1.27) | 0.359 | 0.471 |
| IL-8 (a.u.) | 1.53 (1.27; 1.84) | **<0.001** | **<0.001** | 1.46 (1.21; 1.76) | **<0.001** | **0.001** |
| IL-1β (a.u.) | 1.1 (0.95; 1.27) | 0.185 | 0.327 | 1.05 (0.91; 1.21) | 0.501 | 0.619 |
| IL-18 (a.u.) | 1.06 (0.86; 1.3) | 0.617 | 0.709 | 0.99 (0.82; 1.2) | 0.929 | 0.929 |
| IL-6 (a.u.) | 1.28 (1.11; 1.48) | **0.001** | **0.004** | 1.29 (1.12; 1.49) | **<0.001** | **0.002** |
| TNF-α (a.u.) | 1.13 (0.9; 1.4) | 0.293 | 0.375 | 1.12 (0.92; 1.35) | 0.256 | 0.413 |
| IL-1RA (a.u.) | 1.3 (0.94; 1.79) | 0.112 | 0.235 | 1.2 (0.89; 1.61) | 0.229 | 0.401 |
| sRANKL (a.u.) | 1.12 (0.92; 1.37) | 0.275 | 0.373 | 1.03 (0.85; 1.24) | 0.786 | 0.825 |
| OPG (a.u.) | 1.49 (1.23; 1.8) | **<0.001** | **<0.001** | 1.46 (1.19; 1.8) | **<0.001** | **0.001** |
| **Endothelial dysfunction** |  |  |  |  |  |  |
| sVCAM-1 (a.u.) | 2.9 (1.45; 5.8) | **0.003** | **0.010** | 3.87 (2.07; 7.24) | **<0.001** | **<0.001** |
| sICAM-1 (a.u.) | 1.42 (1.21; 1.67) | **<0.001** | **<0.001** | 1.42 (1.22; 1.67) | **<0.001** | **<0.001** |
| TNFR-I (a.u.) | 1.52 (1.18; 1.96) | **0.001** | **0.006** | 1.46 (1.18; 1.81) | **0.001** | **0.002** |
| **Coagulopathy** |  |  |  |  |  |  |
| PAI-1 (a.u.) | 0.88 (0.58; 1.32) | 0.519 | 0.629 | 0.83 (0.57; 1.19) | 0.305 | 0.427 |
| D-dimer (a.u.) | 1.11 (0.98; 1.26) | 0.105 | 0.235 | 1.09 (0.97; 1.22) | 0.131 | 0.291 |
| **Angiogenesis/Fibrosis** |  |  |  |  |  |  |
| VEGF-A (a.u.) | 1.28 (0.96; 1.7) | 0.096 | 0.235 | 1.19 (0.92; 1.56) | 0.190 | 0.363 |
| VEGF-R1 (a.u.) | 1.14 (0.95; 1.36) | 0.167 | 0.320 | 1.05 (0.88; 1.24) | 0.586 | 0.650 |
|  |  |  |  |  |  |  |
|  | **HIV/HCV-coinfected patients** | | | | | |
|  | **Unadjusted GLM** | | | **Adjusted GLM** | | |
|  | **AMR (95%CI)** | ***p*-value** | ***q*-value** | **AMR (95%CI)** | ***p*-value** | ***q*-value** |
| **Bacterial translocation** |  |  |  |  |  |  |
| LPS (EU/ml) | 0.91 (0.82; 1.02) | 0.102 | 0.407 | 0.91 (0.82; 1.01) | 0.085 | 0.340 |
| LBP (µg/ml) | 1.12 (0.97; 1.28) | 0.114 | 0.407 | 1.12 (0.98; 1.28) | 0.097 | 0.340 |
| sCD14 (µg/ml) | 0.96 (0.82; 1.12) | 0.610 | 0.739 | 0.98 (0.84; 1.14) | 0.806 | 0.846 |
| FABP-2 (ng/ml) | 1 (0.95; 1.05) | 0.884 | 0.924 | 1 (0.95; 1.05) | 0.969 | 0.969 |
| **Inflammatory response** |  |  |  |  |  |  |
| IP-10 (a.u.) | 1.06 (0.98; 1.15) | 0.124 | 0.407 | 1.06 (0.98; 1.14) | 0.157 | 0.375 |
| MCP-1 (a.u.) | 1.02 (0.93; 1.11) | 0.698 | 0.803 | 1.02 (0.93; 1.11) | 0.708 | 0.802 |
| IL-8 (a.u.) | 1.06 (0.94; 1.19) | 0.340 | 0.653 | 1.12 (0.99; 1.26) | 0.075 | 0.340 |
| IL-1β (a.u.) | 1.06 (0.91; 1.24) | 0.451 | 0.692 | 1.07 (0.92; 1.23) | 0.400 | 0.581 |
| IL-18 (a.u.) | 1.02 (0.95; 1.1) | 0.572 | 0.730 | 1.02 (0.95; 1.1) | 0.547 | 0.718 |
| IL-6 (a.u.) | 1.16 (1.08; 1.24) | **<0.001** | **0.002** | 1.15 (1.07; 1.24) | **<0.001** | **0.007** |
| TNF-α (a.u.) | 1.05 (0.92; 1.19) | 0.500 | 0.718 | 1.08 (0.95; 1.22) | 0.233 | 0.464 |
| IL-1RA (a.u.) | 1.1 (1.01; 1.21) | **0.046** | 0.353 | 1.11 (1.01; 1.22) | **0.026** | 0.179 |
| sRANKL (a.u.) | 1.07 (0.91; 1.26) | 0.420 | 0.692 | 1.13 (0.96; 1.33) | 0.141 | 0.375 |
| OPG (a.u.) | 1.07 (0.95; 1.19) | 0.257 | 0.565 | 1.06 (0.95; 1.18) | 0.311 | 0.545 |
| **Endothelial dysfunction** |  |  |  |  |  |  |
| sVCAM-1 (a.u.) | 1.2 (0.88; 1.63) | 0.240 | 0.565 | 1.19 (0.89; 1.61) | 0.243 | 0.464 |
| sICAM-1 (a.u.) | 1.16 (1.05; 1.28) | **0.004** | **0.047** | 1.18 (1.07; 1.3) | **0.001** | **0.007** |
| TNFR-I (a.u.) | 1.06 (0.96; 1.17) | 0.270 | 0.565 | 1.05 (0.95; 1.16) | 0.339 | 0.547 |
| **Coagulopathy** |  |  |  |  |  |  |
| PAI-1 (a.u.) | 1.03 (0.93; 1.14) | 0.564 | 0.730 | 1.02 (0.93; 1.13) | 0.669 | 0.802 |
| D-dimer (a.u.) | 1.05 (0.99; 1.1) | 0.078 | 0.407 | 1.04 (0.99; 1.09) | 0.161 | 0.375 |
| **Angiogenesis/Fibrosis** |  |  |  |  |  |  |
| VEGF-A (a.u.) | 0.99 (0.88; 1.11) | 0.846 | 0.924 | 0.98 (0.87; 1.1) | 0.726 | 0.802 |
| VEGF-R1 (a.u.) | 0.95 (0.84; 1.08) | 0.450 | 0.692 | 0.95 (0.84; 1.07) | 0.415 | 0.581 |

**Statistics**: Values expressed as arithmetic mean ratio (aAMR) and 95% of confidence interval (95%CI). *P-values* were calculated by GLM models unadjusted and adjusted by the main clinical and epidemiological characteristics (see statistical analysis section). *P-values*, raw *p*-values; *q*-values, *p*-values corrected for multiple testing using the false discovery rate (*FDR*) with Benjamini and Hochberg procedure. The statistically significant differences are shown in bold.

**Abbreviations**: HCV, hepatitis C virus; -1, human immunodeficiency virus type 1; a.u., arbitrary units of fluorescence; sCD14, soluble CD14; LPS, lipopolysaccharide; FABP2, fatty acid-binding protein 2; LBP, lipopolysaccharide binding protein; IL, interleukin; IL-1RA, interleukin-1 receptor antagonist; TNF-α, tumor necrosis factor alpha; IP-10, IFN-γ-inducible protein 10; MCP1, monocyte chemoattractant protein-1; OPG, osteoprotegerin; sRANKL, soluble receptor activator of nuclear factor- kappaB ligand; sVCAM-1, soluble vascular cell adhesion molecule 1; sICAM-1, soluble intercellular cell adhesion molecule 1; sTNF-R1, soluble tumor necrosis factor receptor 1; PAI-1, plasminogen activator inhibitor-1; VEGF-A; vascular endothelial growth factor A; sVEGF-R1, soluble receptors for vascular endothelial growth factor.
